# Phacoemulsification: Proposals for Improvement in Its Application

**DOI:** 10.3390/healthcare9111603

**Published:** 2021-11-22

**Authors:** Marta Benítez Martínez, David Baeza Moyano, Roberto Alonso González-Lezcano

**Affiliations:** 1 Department of Chemistry and Biochemistry, Campus Montepríncipe, Universidad San Pablo CEU, 28668 Alcorcón, Madrid, Spain; m.benitez6@usp.ceu.es (M.B.M.); baezams@ceu.es (D.B.M.); 2 Arquitecture and Design Department, Escuela Politécnica Superior, Campus Montpríncipe, Universidad San Pablo CEU, 28668 Alcorcón, Madrid, Spain

**Keywords:** cataract, cataract surgery, endothelial damage, crystalline lens

## Abstract

A cataract is defined as opacity of the crystalline lens. It is currently one of the most prevalent ocular pathologies and is generally associated with aging. The most common treatment for cataracts is surgery. Cataract surgery is a quick and painless process, is very effective, and has few risks. The operation consists of removing the opacified lens and replacing it with an intraocular lens. The most common intraocular lens removal procedure that is currently used is phacoemulsification. The energy applied in this process is generated by ultrasonic waves, which are mechanical waves with a frequency higher than 20 kHz. A great deal of research on the different ways to perform the stages of this surgical procedure and the analysis of the possible side effects of the operation has been published, but there is little information on the technical characteristics, the intensities applied, and the use of ultrasound-emitting (U/S) equipment for cataract removal. More studies on the method and depth of absorption of ultrasonic waves in our visual system when performing the phacoemulsification procedure are needed. It would be advisable for health authorities and medical professionals to develop guidelines for the handling and use of ultrasonic wave-emitting equipment, such as those that exist for ultrasound and physiotherapy. This could help us to reduce undesirable effects after the operation.

## 1. Introduction

### 1.1. Phacoemulsification

Cataracts are defined as the opacity of the crystalline lens and are one of the most prevalent ocular pathologies today, particularly age-related cataracts. They are the leading cause of vision loss [1] and visual impairment around the world (43% and 33%, respectively) [2].

The treatment of this pathology consists of the surgical removal of the opaque crystalline lens and its replacement by an intraocular lens. Cataract surgery is currently a quick, painless, highly effective, and low-risk procedure [3]. About 90% of operated patients are able to see better after the operation [4], which can be undertaken at any stage of cataract development [2].

Phacoemulsification is currently the most widely used surgical method for intraocular lens extraction and is one of the safest and most effective procedures for this purpose [5,6]. The word comes from the Greek φακος (phakos), an ancient technical prefix that alludes to a lens and was used in medicine in ancient times to refer to the crystalline lens. The technique was developed and patented by Dr. Kelman [2] and Anton Banko [7] in 1967.

It consists of breaking up the clouded lens with the help of ultrasound and extracting the resulting broken fragments. Beforehand, the capsule is opened with a micro incision using a scalpel. Phacoemulsification can be used at any stage of cataract development. In place of the extracted lens, a lens with appropriately selected optical parameters is implanted [8].

The resulting advantage of rapid patient mobilisation and visual rehabilitation has established the current well-deserved popularity of phacoemulsification in cataract surgery [9].

One of the main advantages of this technique is that a small incision is usually self-sealing, which effectively shortens the recovery period. [1]

Phacoemulsification cataract surgery usually begins with topical anaesthesia by instilling proparacaine drops on the ocular surface [10]. It can be divided into four stages:

(a) Corneal incision: the ideal incision should be small [7], astigmatically neutral and suture-free. It can be made through an incision in the scleral tunnel or in the clear cornea. [11] The clear corneal incision, which is placed in front of the limbal vascular arcade, is the most commonly used by ophthalmologists due to its self-sealing capability and the implantation of a foldable IOL [12]. The size of the incision, which is about 2.8 mm wide as standard, with options up to 2 mm and smaller, usually depends on the size of the phaco probe [13].

(b) Capsulorhexis: A procedure aimed at disintegrating the crystalline tissue. Ultrasonic energy is transmitted through a hand-held probe equipped with a titanium or steel tip. This vibrates and allows the probe to oscillate and act rapidly against the lens mass. Continuous circular capsulotomy (CCC) is the most popular method of emulsification [1]. It provides ophthalmologists with an intact capsular bag for IOL implantation, was introduced by Gimbel and Neuhann in the mid-1980s [14], and provides numerous benefits [8].

(c) Phacosculpture: The lens is fragmented (emulsified) using U/S and removed through the same instrument after fractionation by aspiration. The most common technique for lens extraction is to initially fragment the lens into four quadrants [15] and then aspirate each quadrant separately [11]. This technique generally incorporates four basic steps and begins with deep sculpting of the nucleus until a very thin posterior plate of the nucleus remains. The next step consists of fracturing the nuclear rim and the posterior plate of the nucleus with lateral pressure using a probe and a spatula. The same step is repeated after rotating the core by 90° to break a wedge-shaped section of the core for emulsion. The procedure is completed by emulsifying each square section systematically [11].

(d) IOL implantation: After phacoemulsification, a posterior capsule is left intact to support an IOL, which replaces the clouded lens [11]. The acrylic lens is the most commonly used lens because it has a higher refractive index and retains most of the advantages of the physical properties of the PMMA lens [16]. Specially designed forceps or an injector are used as the insertion device for IOL implantation. Refractive power correction, incorporated in the recently invented multifocal IOL, allows visualisation of both near and far images on the retina [17]. Therefore, patients can benefit from significant vision restoration without the aid of glasses after cataract surgery [11].

### 1.2. Therapeutic Application of Ultrasonic Waves on the Human Body

Phacoemulsification equipment emits ultrasonic waves. Ultrasonic waves are mechanical waves [11] that have the same physical properties as sound waves audible to humans but are transmitted over shorter distances because they have a higher frequency, diffract less, reflect less from flat surfaces, and are more easily absorbed by the air. U/S waves can propagate in the same direction as particles (longitudinal waves) or in a transverse or perpendicular manner. Longitudinal waves are the most important for the medical application of U/S [12]. U/S waves are mechanical waves originating from the vibrations of an elastic body that propagate through a material medium (body tissues), and their frequency exceeds 20 kHz [13].

ALARA (as low as reasonably achievable) is a concept considered for the use of ionizing radiation in a way that is entirely appropriate for the healthy application of U/S in the human body [12].

The Federal Drug Administration (FDA) has decided to shift the responsibility for the potential risks of U/S use to the supplier of the equipment from the healthcare professional. U/S-emitting equipment has to carry “safety index” information, the thermal index (TI) and the mechanical index (MI). The TI gives an approximate idea of what can happen to tissues exposed to the high temperatures that can be reached when receiving U/S [12]. If certain values are exceeded, tissue heating damage occurs [14]. MI describes the resonant behavior of gas bubbles in liquids, which can cause tissue damage by “inertial cavitation” [12]. This index indicates the mechanical damage that can be caused by the device [14]. Both indexes consider the properties of the tissue wherein the U/S is applied and the biophysical processes associated with it [14].

Some of this energy is lost or redistributed through various mechanisms, such as absorption, scattering, viscosity loss, or thermal conduction [15,16]. U/S can have thermal effects on the human body, depending on the energy flowing over a period of time, and mechanical effects, which depend on the pulse amplitude.

When U/S is applied to humans through the skin with a transducer, 90% of the power is deposited within the first 5 cm of the tissue. Essentially all of the incident acoustic power entering through the skin surface is absorbed into the body tissues [17]. The velocity through the tissue depends on the fat, collagen, and water content. Alternative methods for measuring the depth and manner of absorption of U/S by tissues, such as so-called “estimated in situ exposure”, have been developed, but their direct calculation is complicated. An attenuation coefficient of 0.3 dB cm^−1^ MHz^−1^ has been calculated, taking into account propagation through both soft tissues (with slightly higher loss) and fluids (with lower loss) [12].

The absorption of ultrasonic energy depends on its frequency. Higher frequencies (3 MHz) have a more superficial absorption than lower frequencies (1 MHz). It is recommended to use waves at a frequency of 3 MHz to effectively act at depths of up to 4 cm, while 1 MHz waves can effectively reach approximately 12 cm. The U/S scanning technique uses a pulse frequency of 50 Hz (noncontinuous) with a carrier frequency of 1 MHz to penetrate up to about 15 cm [18].

U/S absorption results in the stimulation of blood circulation (vasodilatation), muscle relaxation by the removal of tissue stimulants, increased membrane permeability, increased tissue regeneration (especially with a mechanical effect), increased or decreased peripheral nerve conduction velocity (thermal effect), decreased pain due to improved tissue circulation [19], and increased speed of biochemical reactions due to increased temperature [20].

The effectiveness of a particular U/S apparatus in achieving the proposed objective depends on the quality of the apparatus, the phenomena of absorption and reflection, and the nature of the tissues that the U/S waves pass through [18].

Ultrasonic waves are used in many areas of life, such as in hydrolocation and underwater telecommunication, industry, and medicine [21]. Ultrasound devices are used in medicine to make diagnostic images such as abdominal, pelvic and cardiac ultrasound. They are also used to treat bones, regenerate soft tissue, remove contractures or remove tumors [22,23,24,25]. Lithotrips (removal of stones in the kidney) use waves from 100 KHz to 200 KHz spaced at 1 s [26]. For the cleaning of jewelry, lenses, watches or instruments for surgery, frequencies between 20 kHz and 40 kHz are normally used, and for the cleaning of teeth ultrasound of 1.6 MHz is used [22].

The U/S emitting equipment used for ultrasound can be classified, according to some authors, into three groups: sectorial, the working frequencies of which are usually 3.5 to 5 MHz [24,27,28]; convex, the working frequencies of which are usually 3.5 to 5 MHz; and linear, the working frequencies of which are usually 7.5 and 13 MHz. However, frequencies up to 20 MHz have been used [20,24]. The U/S waves used in ultrasound are of very low intensity (10–50 mW/cm^2^) in order to avoid changes in the media through which they pass [22]. In physiotherapy, the frequencies used are in the range of 0.5 to 5 MHz, and the maximum power flux value specified is 3 W/cm^2^ [29]. The exact energy reaching the target tissue is not known, so it is not possible to determine dose–response curves. Because of this, treatment planning is based on the experience of each physiotherapist [30].

In treatments for hypodermic fat reduction, frequencies of 40 kHz, 1 MHz, and 3 MHz are used for high-intensity focused ultrasound (HIFU) with powers of up to 30 W, 50 W, and 90 W, respectively [18,31,32].

## 2. Results

### 2.1. Complications in the Phacoemulsification Technique

Damage may occur where the scleral corneal cut is made. This can be due to the heat generated by the U/S waves, leading to difficulty in wound healing. This can lead to heat leakage and the induction of high degrees of postoperative astigmatism. The use of excessive ultrasonic power and the loss of adequate fluid flow around the phacoemulsification tip are the main causes of ocular tissue damage after phacoemulsification cataract surgery [33].

Coaxial phacoemulsification causes changes in the surface temperature of the cornea, eye, and orbital cavity [34]. Corneal and scleral burns may be caused by increased ocular surface temperature in the area around the phaco handpiece [35].

Thermography is a useful tool that can be used routinely in the operating room to monitor ocular surface temperature. Some studies have shown that the temperature in the posterior chamber does not change during the operation, regardless of the procedure used, but the temperature in the anterior chamber does [36]. No significant differences in ocular surface temperature were found between healthy phakic and pseudophakic patients one month after cataract surgery, indicating a normalization of temperature [37], but there is a negative correlation between age and ocular surface temperature [38]. Ocular surface temperature does not increase significantly after cataract surgery, suggesting an absence of significant inflammation, and the temperature about one month after cataract surgery is comparable to the temperature before surgery. The negative correlation between age and ocular surface temperature should be of concern in the elderly [34].

Dry eye syndrome increases rapidly after cataract surgery. Thermal imaging reveals that dry or pathologic eyes have a lower temperature than normal eyes. This may be explained by the lower emissivity of the unstable tear film [39]. Increasing patient awareness of the possibility of the clinical symptoms of dry eye syndrome during the first month after surgery should be part of the clinical management of cataract surgery [34].

Endothelial damage associated with the phacoemulsification process may be caused by free radicals [5]. During phacoemulsification cataract surgery, the contents of the aqueous humor are, in most cases, exchanged for an ophthalmic irrigating solution. The irrigating solution is circulated through the eye at a typical rate of 20 to 30 mL/min to prevent heat buildup produced by phacoemulsification and facilitate the surgical procedure. This action also flushes out the physiological antioxidants that normally protect the ocular tissues from free radical damage [40].

Many chemical reactions under the influence of U/S occur in aqueous solutions. Free radicals are thought to be generated when the heat from the implosion of cavitation bubbles causes the decomposition of water [41]. U/S [42] oscillation in an aqueous solution produces OH, which is known to be the most reactive of the various reactive oxygen species and is associated with many pathologic conditions. H_2_ dissolved in irrigation solution reduced corneal endothelial damage during phacoemulsification. This suggests that a considerable part of the corneal endothelial damage caused during phacoemulsification is due to oxidative stress, and that H_2_ is useful in clinical phacoemulsification. [43]

Human corneal endothelial cells (HCECs) are responsible for maintaining corneal transparency by regulating the hydration of the corneal stroma [44]. U/S energy during phacoemulsification may cause mechanical trauma to the corneal endothelium, leading to prolonged postoperative recovery [44]. Possible endothelial cell leakage (ECL) is a major postoperative complication that could [45] lead to later posterior lamellar endothelial keratoplasty or even penetrating keratoplasty [46].

Posterior capsule rupture (PCR) is a common and serious complication of phacoemulsification [47]. PCR usually occurs toward the end of surgery, when the last quadrant of the nucleus is removed. The main reason for this is that the posterior capsule is more exposed in the final stages of the operation [48].

The rupture of the posterior capsule complicates the operation, especially when vitreous prolapse occurs. PCR may also result in the postoperative deterioration of visual acuity. Moreover, without proper treatment, PCR can cause many additional complications, such as retinal detachment, macular edema, uveitis, glaucoma, and intraocular lens (IOL) dislocation [49].

If PCR occurs, it is essential to remove the vitreous and residual nuclear fragments from the wound, anterior chamber, or vitreous cavity by anterior vitrectomy or pars plana. The risk of PCR increases in difficult cases, such as small pupil, pseudoexfoliation (PXF), intraoperative floppy iris syndrome (IFIS), and dense cataracts, such as mature, white, intumescent, or black cataracts [48].

The literature has reported breakage rates ranging from 0.5 to 10%, with lower rates occurring when surgeons are more experienced with the technique [50]. In addition, an increase in complications may also be associated after a period of rest or surgical abstinence by the surgeons [51].

Posterior capsular ruptures place a considerable economic burden on the healthcare system [52]. An improved fluid system greatly stabilizes the anterior chamber. Patients with a foldable IOL tend to have much better outcomes. The foldable IOL can play a similar role as the epinucleus and be used as an ideal tool to reduce the risk of PCR when emulsifying the last nuclear quadrant. Therefore, the foldable IOL can be used to improve surgical safety [48].

With the continued development of phacoemulsification equipment, many cataract surgeons now use a maximum vacuum level to complete surgery quickly. One of the potential complications associated with an increased vacuum level is unacceptable anterior chamber instability, which can be addressed by providing more infusion, resulting in transient elevations in intraocular pressure (IOP) [53].

Numerous studies have found that a 20 mm Hg increase in IOP for 5 min resulted in reduced blood flow to the optic nerve, retina, and choroid in healthy subjects [54]. Acute elevations in IOP for less than 1 min may inhibit the retrograde transport of essential neurotrophins from the brain to the retina [55]. In addition, there may be a causal relationship between elevated IOP associated with cataract surgery and nonarteritic anterior ischemic optic neuropathy (NAION), which can occur within 24 h of surgery [56]. These reports have documented the potential adverse effects of transient IOP fluctuations on retinal and optic nerve function and visual acuity recovery.

Even eyes with normal preoperative values and an uncomplicated course of phacoemulsification may show very elevated IOP values postoperatively, which can cause pain and blurred vision and, rarely, compromise visual function [57].

There is a possibility of developing postoperative macular edema, resulting in suboptimal postoperative vision, in 0.1 to 2% of the healthy population, but it is predominant in patients who have pre-existing diabetic macular edema [58]. Several studies have reported conflicting results regarding corneal changes after phacoemuslification in diabetic and nondiabetic patients [44]. Diabetes mellitus is a risk factor in the development of postoperative macular edema, with a consequent risk of decreased visual outcomes after cataract surgery. A good control of diabetes is necessary to prevent macular thickening and macular edema after phacoemulsification surgery. The recovery of the corneal edema [44] and quantitative changes in the macula [59] in diabetic and nondiabetic eyes after uncomplicated cataract surgery have been evaluated in different studies. No statistically significant differences in CMT between normal subjects and diabetic subjects without diabetic retinopathy were found in the preoperative and early postoperative period after uncomplicated phacoemulsification surgery.

Some authors believe that there are certain prior risks (hardness of nucleus, shorter axial length, or presence of diabetes mellitus) [45] or complications that may occur during or after this type of operation, but it is still a very beneficial technique with a proven success rate [56].

### 2.2. Studies of Different Procedures to Perform the Cataract Operation

The revolution in cataract surgery has never stopped [60]. Surgical techniques have changed dramatically in recent years, with consequent improvements in outcomes and safety [1].

Early intracapsular cataract extraction (ICCE) [61], which was a technique discovered in the 18th century, involves removing the entire lens and capsule through a single incision [62]. Due to lower complication rates with improved surgical techniques, ICCE is now rarely performed [63]. Extracapsularcataract extraction (ECCE) was develop next. This involved removing the lens through an incision, and then the intraocular lens (IOL) is inserted. The incision is large, usually 9–13 mm, in order to perform the removal, and sutures are needed [62]. Next came manual small-incision cataract surgery (MSICS):,where instead of a large incision, MSICS used a scleral tunnel that could be self-sealed. The much smaller external incision (6.5 mm to 7 mm) with a larger internal incision (9 mm to 11 mm) allows a natural [62]. And by the current phacoemulsification (PCS) discussed above.

Surgical techniques have changed dramatically in recent years, with consequent improvements in outcomes and safety. Femtosecond laser platforms that can accurately and reproducibly perform the key steps of cataract surgery, such as corneal incisions, capsulotomy, and lens fragmentation, are now available [1].

Cataract surgeons are adopting femtosecond technology to perform laser capsulotomy, lens fragmentation [56], clear corneal incisions, and limbal relaxing incisions. Femtosecond lasers are able to perform these surgical steps with great precision and reproducibility [64]. Laser energy can be directed to corneal tissues to create self-sealing corneal and limbal relaxing incisions. It can also be directed at the anterior lens capsule to create perfectly round well-centered capsulotomy buttons of precise diameter [64].

In an analysis of a total of 989 eyes and nine randomized, controlled trials, a statistically significant improvement for FLACS (femtosecond laser cataract surgery) over MCS (manual cataract surgery) was found in terms of mean phacoemulsification energy and effective phacoemulsification time; however, no difference in surgical complications was found [65].

A review of sixteen randomized controlled trials (RCTs) was not able to determine the equivalence or superiority of laser-assisted cataract surgery compared to standard manual phacoemulsification for our chosen outcomes because of the low to very low certainty of the evidence available from these studies [1].

Our current objective is to perform comparative studies on certain parameters to see whether they can be improved when the femtosecond laser is used in the phacoemulsification technique [66].

We compared the amount of ultrasound energy and the volume of irrigation [6] (measuring the effective phacoemulsification time (EPT), which indicates the ultrasound energy, and the use of balanced salt solution (BSS), which indicates the volume of irrigation) to indirectly estimate endothelial damage. We found a reduction in the effective phacoemulsification time, but no influence on the use of BSS, which was able to indirectly estimate endothelial damage [3].

No changes in corrected distance visual acuity and no significant improvements or differences in patient-reported health and safety outcomes were found after 12 months of follow-up [66]. The mean absolute refractive error was not significantly different either [67].

In an evaluation of optical quality recovery and various macular thickness changes, where modulation transfer function (MTF), point spread function (PSF), and dysfunctional lens index (DLI) were measured using a ray-tracing aberrometer (iTrace), it was concluded that FLACS (femtosecond laser) was safe and did not cause a significant increase in macular thickness [68]. Following the criteria to analyze the success rate of surgery (defined as a composite of four outcomes: no serious perioperative complications, a best corrected visual acuity of 0–0 LogMAR (logarithm of the minimum angle of resolution), an absolute refractive error of 0–75 diopters or less, and an unchanged postoperative corneal astigmatism power), no significant differences in success rate were identified between femtosecond vs. traditional scalpel capsule opening in phacoemulsification [67].

Laser cataract surgery, regardless of the possible improvements that could be achieved in visual acuity outcomes and complication rates, currently has a high cost to the patient when compared to cost-effectiveness benchmarks and other medical interventions, such as the traditional phacoemulsification technique [69]; thus, its use is limited [70].

Regarding the use of femtosecond laser pretreatment to perform the incision, some researchers have stated that it has not been proven to be harmful [71], while others have stated that there is little certainty [1] of improvement when using the laser, that it does not increase the endothelial damage caused by cataract surgery, and that it does increase the damage if the incision is made manually, as it is harmful for eyes that have low endothelial cell values before undergoing surgery [72]. Femtosecond laser pretreatment for cataract surgery was associated with a significant reduction in early postoperative corneal edema and endothelial cell loss compared with conventional phacoemulsification; however, the difference diminished with time [73].

### 2.3. Risk Factors in Cataract Surgery

There are contraindications that make certain patients unsuitable for this type of intervention. Pathologies can vary from patients with very high astigmatism to autoimmune diseases. Diabetic patients are more likely to develop cataracts and there is a decrease in visual outcomes after cataract surgery [64]. Patients with dry eye syndrome (DED) may have very different IOL power calculations, irregularities and corneal asymmetries [74]. The decision whether or not to perform surgical treatment is a difficult one, as the ophthalmologist must also take into account the patient’s priorities and whether the postoperative outcome meets his or her expectations [75].

Posterior capsule rupture (PCR) is the most common major intraoperative complication observed during cataract surgery. Rupture rates range from 0.5% to 10% [76]. If PCR occurs, it is essential to remove the vitreous and residual nuclear fragments from the wound, anterior chamber or vitreous cavity by anterior vitrectomy. Failure to do so increases the risks of leakage, infection or vitreous traction that may lead to cystoid macular oedema or retinal detachment [51].

The pathogenesis of elevated IOP is multifactorial, involving factors such as inflammation, haemorrhage, pigment dispersion and others. If left untreated, uncontrolled postoperative IOP spikes can lead to pain, corneal oedema, and glaucomatous optic nerve damage. The pattern of IOP change shows a gradual peak increase in the first postoperative hours at four and six hours followed by a normalisation of pressure a day later [77].

There is a reduction in temperature after surgery with the greatest decrease after 15 days, then it rises again approximately one month later to previous values.

To minimise the risk of infection after surgery, proper ocular hygiene must be maintained, and the prescribed medication must be used correctly. Other common symptoms may include inflammation, pain, redness, loss of vision, swelling or bleeding in the eye [78].

Other more serious complications may include vitreous detachment [76], IOL dislocation [79], transient and persistent postoperative ptosis [80] or retinal detachment. The latter does not cause pain. Early treatment may prevent permanent vision loss [81].

### 2.4. Ultrasonic Frequency and Power Used by the Phacoemulsification Equipment

The needle used in the phacoemulsification process passes through the aqueous and nucleus, which have little resistance [7]. Together with the mechanical effect generated by moving the phaco handpiece over the lens material [20,82], small bubbles are formed in the liquid environment in which the U/S is applied. A large energy release occurs as the bubbles collapse due to the physical phenomenon called cavitation [6,7]. Cavitation could be defined as the growth, oscillation, and collapse of micrometer-sized bubbles in liquids under the influence of an acoustic field [7].

Early phacoemulsification machines were based on longitudinal ultrasonic vibrations but had several drawbacks, such as the repulsion of lens fragments from the phaco handpiece [83]. Recently, the use of nonlongitudinal phacoemulsification has become widespread. This technique consists of using torsional and transverse ultrasonic vibrations and their various combinations for cataract destruction. These modifications make it possible to reduce repulsion and increase the tracking and cutting ability of the ultrasonic handpiece, meaning that the ultrasonic energy is used more optimally [11]. The torsional technique provides a faster visual recovery than the linear technique. However, no statistically significant differences were found in visual acuity, endothelial cell loss, postoperative corneal edema, or postoperative swelling between the two techniques when comparing preoperative conditions and the conditions on postoperative day 28 [84].

One technique used in phacoemulsification cataract surgery is known as torsional Ozil, a system developed in 2005 in which ultrasonic waves with a frequency of 32 kHz are used continuously or in bursts [72]. This technique is based on the rotational oscillation of the phaco handpiece. The side-to-side motion reduces the repulsion of the lens fragments, thus minimizing the side effects of the procedure [85].

The ultrasonic frequencies currently used are between 35,000 and 45,000 cycles per second (hertz), as these are the most efficient for nuclear emulsification. Lower frequencies appear to be less efficient and higher frequencies create excess heat [7].

In a review conducted on 2802 selected articles, 14,567 eyes from 15 randomized controlled trials and 22 observational cohort studies were included [86], as were references for some equipment used. The frequency and power of the U/S waves generated by these devices and links to their user manuals are shown in Table 1.

The higher the micropulse is, the less ultrasonic energy is needed [87]. Several researchers have agreed that a longer phacoemulsification time, higher phacoemulsification energy, high vacuum, and lower phacoemulsification frequency are independent parameters for increasing CDE loss [43,88].

The effective phacoemulsification time (EPT) [3] or ultrasound time (UST) are parameters used for measuring the U/S exposure time. The mean cumulative dissipated energy (CDE) power indicates the mean percentage of power spent during the UST. The CDE was calculated in accordance with the guidelines of the phaco unit manufacturer and with the recommendations of previous studies [85].

Some authors have proposed an increase in vacuum and suction [73] or the use of three-dimensional U/S to achieve greater efficiency in the phacoemulsification process [11].

### 2.5. The Importance of Virtual Reality (VR) Simulators

The evaluation of surgical tasks performed on virtual reality (VR) simulators showed a significant difference in performance before and after training [8] and between resident ophthalmologists in training and staff ophthalmologists [89].

In performing the simulation, subjects with more experience obtained significantly higher scores in the four main procedures: corneal incision (CI), capsulorhexis (C), phacoemulsification (P), and intraocular lens implantation (IOL) [10]. The complication rate was significantly higher (6.3%) for residents in training, with the IOP complication rate elevation being the most significant [89].

Augmented VR simulators have the potential to be used as a feasible competency assessment tool for the four complete major surgical procedures and provide a good training method [8].

## 3. Discussion and Conclusions

Despite phacoemulsification being the most widely used technique for cataract extraction, the authors of this review found few publications detailing the power and frequency emitted by phacoemulsification equipment.

Medical treatment publications (ultrasound, physiotherapy, etc.) show the frequency and absorption shape of the applied waves (MHz), but there are no completely uniform criteria. There is a lack of information regarding the form of absorption and the depth of penetration into the human body in general for ultrasonic waves with a frequency in the kHz, which is the frequency used in phacoemulsification. Further research on the absorption of U/S applied with a kHz frequency should be carried out.

While the user manuals for phacoemulsification equipment usually provide information regarding the power used in the diathermy and laser processes, it is not easy to find the power of the U/S that can be applied in the phaco process in such a clear way. Without knowledge of the power, we cannot calculate the intensity applied and therefore the possible dosage that the patient may receive from U/S waves. Studies of different procedures to perform the cataract operation are explained in this article. There are many published studies on the positive consequences of the use of the femtosecond laser. This section serves to contrast the lack of equivalent studies on the possible effects of ultrasound on the visual system after its use.

It would be interesting to consider the possibility of developing an instruction manual for the use of U/S-emitting equipment to perform phacoemulsification, showing how the equipment should be applied and considering the intensity applied for calculation recommended doses and safety parameters like those used in other branches of medicine (IM and IT) in which ultrasonic waves are applied for therapeutic purposes. Virtual reality simulators could be useful for this process.

We suggest that the phaco handpiece should be applied horizontally, never in the direction of the central area of the retina. Special care should be taken not to put it at any time in the direction of the optic nerve or the macula. The handyman must always be in continuous movement without being more than one or two seconds pointing to a specific position. Attempts should be made to set a maximum power to be applied by the health authorities.

Simulation equipment to train ophthalmologists in the phacoemulsification process may be an option for improvement in this procedure. The simulator would have to penalize the operator of the trial if it is aimed at the macular area, optic nerve, or keeps more than 1 or 2 s off the phacoemulsifier. It would be possible to modulate the power and time of the process

## Figures and Tables

**Table 1:** The frequency and power of U/S devices and links to the user manuals for the devices.

Device	Phacoemulsification Frequency	Phaco Power	Device Use Manual
MEGATRON S4 HPS (Geuder Group, Heidelberg, Germany)	27–55 kHz	% use	Megatron (Geuder Group, Heidelberg, Germany), megaTRON S4 HPS Microsurgical System: Geuder AG
STELLARIS B + L	28.5 kHz	35 W	Stellaris (Bausch & Lomb, Inc., Rochester, NY, USA.) Stellaris PC. Vision Enhancement System: https://www.bausch.com/Portals/77/-/m/BL/Global/dfu/110017276EN_REVA.pdf (accessed on 25 April 2021)]
Infinity (Alcon, Inc., Irvine, CA, USA)	35–41 kHz	35 W	OcuScan^®^ RxP Measuring System OPERATOR’S MANUAL: http://www.frankshospitalworkshop.com/equipment/documents/ophthalmology/user_manuals/Alcon%20Infiniti%20-%20Operator%27s%20manual.pdf (accessed on 1 May 2021)]
Constellation (Alcon, Inc., Irvine, CA, USA.)	34–42 kHz ± 10%	Not found	212-1 Constellation Vision System User Manual NGVS Book 1.indb Alcon Research [fccid.io]
SERIES 20000™* LEGACY (Alcon, Inc., Irvine, CA, USA)	38 kHz	% use	SERIES 20000™* LEGACY ^®^ SERVICE MANUAL: http://www.frankshospitalworkshop.com/equipment/documents/ophthalmology/service_manuals/Alcon_Phaco_machine_Series_20000_Legacy_-_Service_manual.pdf (accessed on 14 May 2021)

## Data Availability

Not applicable.
